# The utility of sonographic signs to diagnose simple and complicated appendicitis in children

**DOI:** 10.1007/s00383-023-05397-y

**Published:** 2023-02-11

**Authors:** Lauren Tong, Ramesh M. Nataraja, Keith VanHaltren, Tania H. Sulaksana, Toby I. Vinycomb, Maurizio Pacilli

**Affiliations:** 1 Department of Paediatrics, School of Clinical Sciences at Monash Health, Faculty of Medicine, Nursing and Health Sciences, Monash University, Melbourne, VIC Australia; 2 Department of Surgery, School of Clinical Sciences at Monash Health, Faculty of Medicine, Nursing and Health Sciences, Monash University, Melbourne, VIC Australia; 3 Department of Paediatric Surgery, Monash Children’s Hospital, Level 5, 246 Clayton Road, Clayton, Melbourne, VIC 3168 Australia; 4 Department of Paediatric Radiology, Monash Children’s Hospital, Melbourne, VIC Australia

**Keywords:** Ultrasound scan, Pediatric appendicitis, Sonographic sign, Simple appendicitis, Complicated appendicitis, Diagnostic accuracy

## Abstract

**Background:**

Acute appendicitis is classified into simple (SA) and complicated (CA). Ultrasound scans (USS) can be useful in clinically equivocal cases, by visualising primary and secondary signs. This study explores the utility of sonographic signs to diagnose and differentiate appendicitis in children.

**Methods:**

Single-centre retrospective cohort study over a 2-year period. Consecutive USS for suspected appendicitis were included; sonographic signs were extracted from standardised institutional worksheets. USS results were compared with pre-defined intraoperative criteria for SA and CA, confirmed with histological analysis. Data are reported as median [interquartile range], percentages (number), area under the curve (AUC), conventional diagnostic formulae and adjusted odds ratios following multiple logistic regression (*p* < 0.05 considered significant).

**Results:**

A total of 934 USS were included, with median age 10.7 [8.0–13.4] years, majority were female (54%). One quarter (*n* = 226) had SA, 12% (*n* = 113) had CA, 61% (*n* = 571) had no appendectomy and 3% (*n* = 24) had negative appendicectomy. Appendix visualisation rate on USS was 61% (*n* = 569), with 62% (*n* = 580) having a conclusive report. Sonographic signs suggesting appendicitis included an appendiceal diameter > 7 mm (AUC 0.92, [95% CI: 0.90–0.94]), an appendicolith (*p* = 0.003), hyperaemia (*p* = 0.001), non-compressibility (*p* = 0.029) and no luminal gas (*p* = 0.004). Secondary sonographic signs included probe tenderness (*p* < 0.001) and peri-appendiceal echogenic fat (*p* < 0.001). Sonographic signs suggesting CA over SA comprised a diameter > 10.1 mm (AUC 0.63, [95% CI: 0.57–0.69]), an appendicolith (*p* = 0.003) and peri-appendiceal fluid (*p* = 0.004).

**Conclusion:**

Presence of specific sonographic signs can aid diagnosis and differentiation of simple and complicated appendicitis in children.

**Supplementary Information:**

The online version contains supplementary material available at 10.1007/s00383-023-05397-y.

## Introduction

Acute appendicitis is the most common surgical emergency in children. It can be classified into simple (SA) and complicated appendicitis (CA); the definition of CA remains unclear in the literature, with the presence of a visible hole, diffuse fibrinopurulent exudate, intra-abdominal abscess, and extraluminal fecalith often regarded as findings of CA[1]. Diagnosis is usually clinical, although it may be elusive in children, with frequent atypical presentations and communication limitations. Clinical risk scores, such as the Alvarado score, are most commonly used to stratify patients in emergency department (ED) settings, although clinician gestalt performs similarly [2]. Laboratory tests lack required sensitivity and specificity, with levels fluctuating with symptom duration [3, 4].

Consequently, imaging is performed in equivocal presentations, with abdominal ultrasound scan (USS) widely regarded as the initial modality of choice. USS is convenient and lacks ionising radiation or need for sedation/anesthesia; however, accuracy is multifactorial being highly operator dependent, with frequent inconclusive reports [5, 6]. Appendicitis can display primary sonographic signs of appendiceal inflammation, such as an enlarged diameter; and secondary (surrounding) signs such as echogenic fat, when the appendix is non-visualised.

The accuracy of USS to diagnose appendicitis has been benchmarked in paediatric cohorts, however, the ability to differentiate SA from CA has not been widely studied [7]. SA and CA have distinct clinical courses, and whether USS can reliably distinguish them is yet to be determined. This study aims to assess the utility of sonographic signs for diagnosing and differentiating acute appendicitis in children.

## Methods

### Setting

Our tertiary paediatric surgical centre has a dedicated paediatric radiology department with rotating sonographers who perform USS, and paediatric radiologists (on-call 24-h) that report them. We utilise a Samsung RS85 ultrasound, with multiple convex (C3-10, C4-9) and linear (L3-12) transducers for each abdominal examination. When scanning the appendix, Puylaert’s graded compression technique along with various visualisation strategies are used [8]. These include left lateral decubitus positioning, posterior manual compression, micturition, and respiration; with a second sonographer requested where feasible. Scanning is performed using a standardised sonographer worksheet (Supplementary Material). Operators must indicate appendiceal visualisation and confirmation of blind-ending, as well as four primary sonographic signs: maximal diameter (mm), hyperaemia, compressibility, absent luminal gas; and three secondary signs: probe tenderness, peri-appendiceal fluid and peri-appendiceal echogenic fat.

### Study design

All children presenting to the Emergency Department (ED) with a principal complaint of abdominal pain were retrospectively considered for inclusion between January 2017 and May 2019. Consecutive USS reports including the terms “appendix” or “appendicitis” were screened. To verify they were performed for suspected appendicitis, correlation with the electronic hospital records and the clinical indications were reviewed. USS were excluded if primarily investigating chronic non-specific abdominal pain, intussusception, or a known appendiceal mass. Children with previous appendicectomy were excluded.

USS were grouped into four operative outcomes: No Appendicectomy (NoA), Negative appendicectomy (NegA), SA and CA. NoA was confirmed via clinical follow-up for 12 months, NegA was confirmed on histology in the absence of acute inflammation. SA was defined as non-perforated, incorporating inflammatory changes or gangrenous appendices. CA was intraoperatively defined as macroscopic perforation, intraperitoneal appendicolith or four-quadrant pus [1]. CA also included clinically or radiologically detected appendiceal abscess or mass. If a child had undergone two USS before intervention, the former was classified as NoA, with the latter in the appropriate operative category; assuming this aided the decision for surgery. When an intraoperative result suggested SA, and histology demonstrated CA; intraoperative results were utilised. This reflects clinical management and acknowledges potential iatrogenic perforation during appendicectomy. Clinical follow-up was for 12 months.

### Data analysis

Data were analysed using GraphPad Prism (Version 8.4.3, San Diego, U.S.A) and MedCalc (Version 19, Ostend, Belgium). Data are reported as median [interquartile range] and numbers (percentages). Following D’Agostino-Pearson normality test, Kruskal–Wallis one-way analysis of variance compared continuous variables between all operative groups, with Dunn’s multiple comparisons post-hoc test. Chi-squared was performed for binary outcomes. Area under the curve (AUC) analysis determined the optimal threshold for categorising the appendix by diameter. Forced entry of variables into multiple logistic regression analysis was undertaken, reporting adjusted odds ratios to find significant sonographic predictors of the diagnosis and classification of appendicitis. Diagnostic utility was measured with conventional formulae, sensitivity, and specificity, with 95% confidence intervals (CI) computed using Wilson-Brown’s test. We conservatively regarded ‘positive’ USS as radiologist concluded appendicitis, with ‘negative’ USS including equivocal reports as well as concluded normal. To determine accuracy for diagnosing CA over SA, only the USS which concluded appendicitis were used. A two-sided *p*-value < 0.05 was considered significant.

### Human research ethics committee

HREC approval was granted before commencement (Local reference: RES-20-0000250Q-63608), with informed consent waived.

## Results

**Fig. 1:**
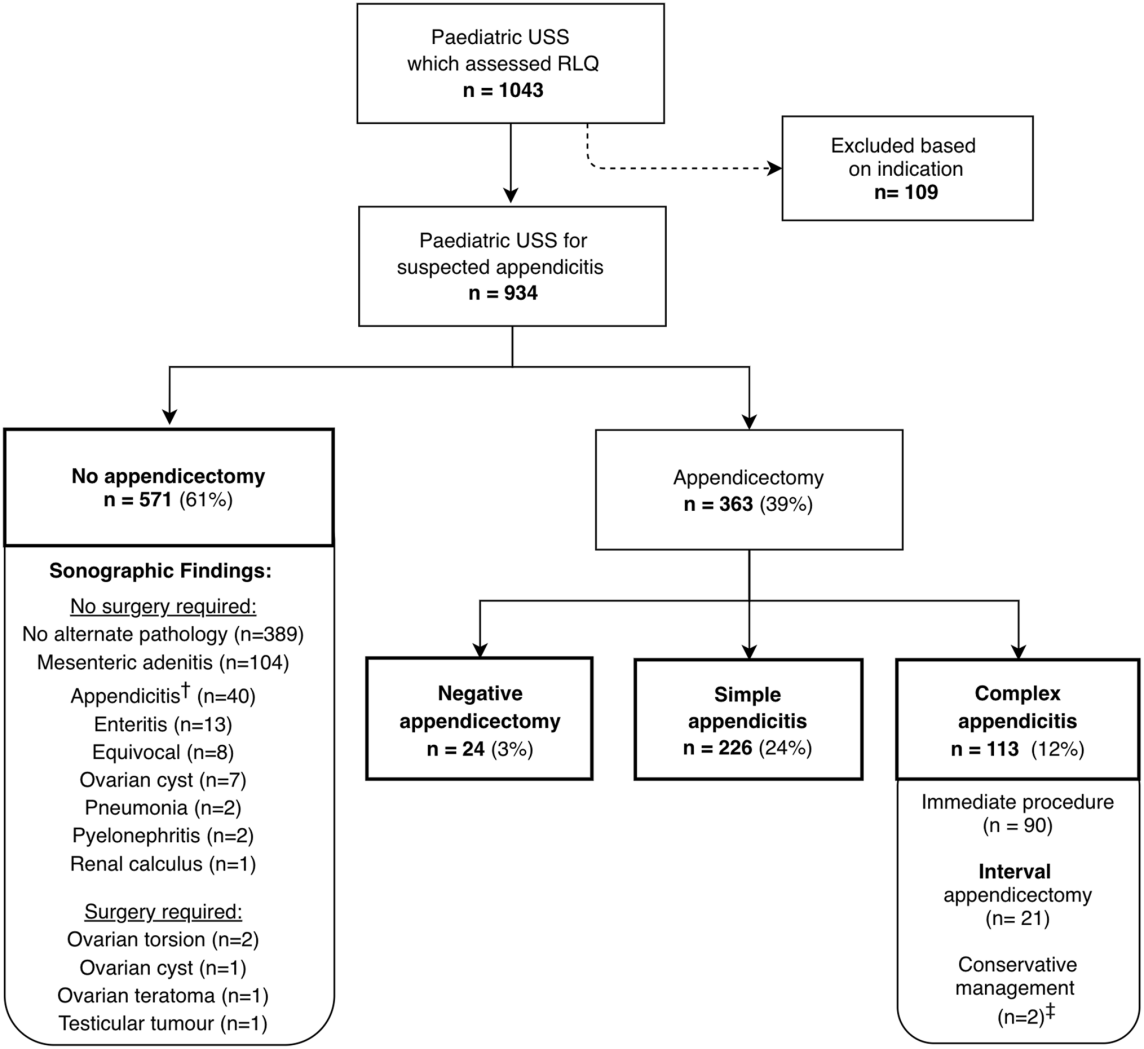
Flow diagram of study population. ^†^Forty USS concluded appendicitis; however, most children (*n* = 36) symptomatically improved on admission and were subsequently discharged; four children had repeat USS and underwent appendicectomy. ^‡^Two children had evidence of an appendiceal abscess and underwent percutaneous drainage and antibiotic management, without interval appendicectomy

Over the study period, 12,185 children presented with a primary complaint of abdominal pain to our ED network sites. The paediatric surgery department performed 705 appendicectomies. Paediatric radiology completed 1,043 USS which assessed the appendix (Fig. 1); with 14 X-Rays, three CT scans and one MRI performed. Following exclusion of USS ordered for indications other than suspected appendicitis (*n* = 109), the final cohort comprised 934 USS in 877 children.

### Cohort characteristics

The median age was 10.7 years [8.0–13.4], with 54% being female. Children with CA were significantly younger than children with SA (*p* < 0.001); 9 [6–13] and 12 [9–14] years, respectively (Table 1). Females comprised a significant majority (92%) of the NegA group (*p* < 0.001).

**Table 1:** Demographics of study population

Demographic	All scans (*n* = 934)	NoA (*n* = 571)	NegA (*n* = 24)	SA (*n* = 226)	CA (*n* = 113)	*P*-value
Age (years)	10.7 [8.0–13.4]	10.4 [7.7–13.3]	11.0 [9.5–14.2]	11.7 [9.3–13.8]	9.0 [6.0–12.7]	** < 0.001**
0– < 6: *n* (%)	114 (12.2)	75 (13.1)	1 (4.2)	12 (5.3)	26 (23.0)	
6– < 12: *n* (%)	480 (51.4)	300 (52.5)	12 (50.0)	114 (50.4)	54 (47.8)	
12–19: *n* (%)	340 (36.4)	196 (34.3)	11 (45.8)	100 (44.3)	33 (29.2)	
Sex *n* (%)						** < 0.001**
Male	432 (46.3)	252 (44.1)	2 (8.3)	122 (54.0)	56 (49.6)	
Female	502 (53.7)	319 (55.9)	22 (91.7)	104 (46.0)	57 (50.4)	

Bold represents significant *P*-values

Data reported as median [interquartile range], number (percentage). *P*-values computed using Kruskal–Wallis test

*NoA* No Appendicectomy, *NegA* Negative Appendicectomy, *SA* Simple appendicitis, *CA* Complicated appendicitis

Most USS did not result in a surgical intervention, with 61% having NoA (*n* = 571), 3% had a NegA (*n* = 24), 24% had SA (*n* = 226) and 12% had CA (*n* = 113). Appendix visualisation rate on USS was 61% (*n* = 569), with 62% (*n* = 580) having a conclusive report (including where other diagnoses were sonographically identified *n* = 137).

The overall NegA rate in our study period was 5.5% (39/705). No significant difference in NegA rate was found between children with (6.7%) or without (4.4%) a pre-operative scan; *p* = 0.18.

Time from USS to surgery was slightly longer in the SA group comparing to the CA group: 11.27 [3.0–20.0] hours vs 6.8 [4.5–22.2] hours; *p* = 0.05.

### Sonographic signs

**Fig. 2:**
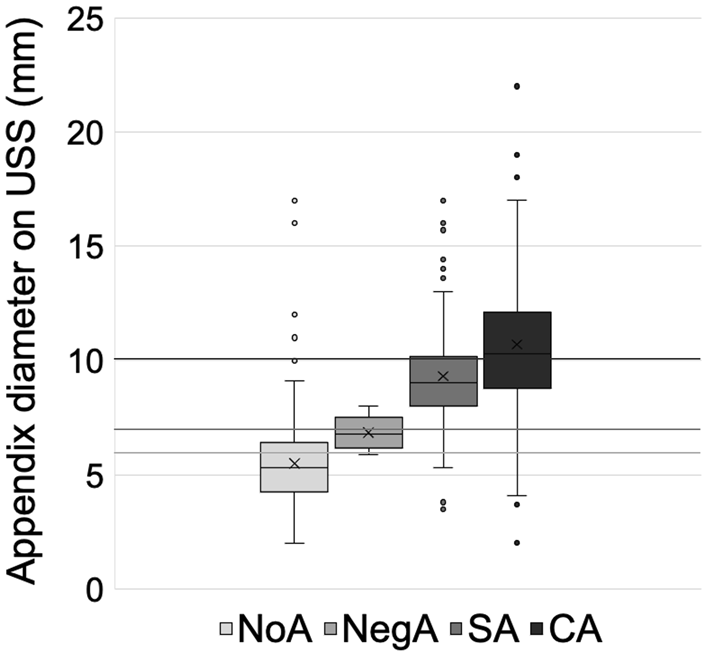
Sonographic appendiceal diameter across groups. Box and whisker plot of maximal appendiceal diameter (mm) across groups, with inserted 6 mm, 7 mm and 10.1 mm thresholds (*p* < 0.001 between groups using Kruskal–Wallis and Dunn’s multiple comparisons). 7 mm threshold: AUC 0.92 [95% CI: 0.90–0.94], sensitivity 86%, specificity 86%. 10.1 mm threshold: AUC 0.63 [95% CI: 0.57–0.69], sensitivity 54%, specificity 75%. *USS* Ultrasound Scan, *NoA* No Appendicectomy, *NegA* Negative Appendicectomy, *SA* Simple appendicitis, *CA* Complicated appendicitis

Sonographic signs which suggested appendicitis included an appendiceal diameter above 7 mm (AUC 0.92, [95% CI: 0.90–0.94] sensitivity 86%, specificity 86%) (Fig. 2), presence of an appendicolith (*p* = 0.003), hyperaemia (*p* = 0.001), non-compressibility (*p* = 0.03), and no luminal gas (*p* = 0.004) (Table 2). When these latter four significant variables were grouped, the regression model correctly classified 80% of cases with AUC 0.87 [95% CI: 0.83–0.90]. Secondary signs included probe tenderness (*p* < 0.001) and presence of peri-appendiceal echogenic fat (*p* < 0.001). When these two variables were grouped, the regression model correctly classified 82% of cases with AUC 0.85 [95% CI: 0.82–0.89]. Overall, we found USS had diagnostic accuracy of 89%, with sensitivity 84.7% [CI: 80.4–88.1] and specificity 92.1% [CI: 90.0–94.0].

**Table 2:** Utility of sonographic signs to diagnose appendicitis

Sonographic sign	Appendicitis (*n* = 339) %	Other (*n* = 595) %	Sensitivity %, (95% CI)	Specificity %, (95% CI)	Adj. Odds ratio	*P*-value
Blind ending (*n* = 537)	89.5	92.4	89.5 (85–93)	7.6 (5–12)	0.88	0.06
Appendicolith (*n* = 523)	30.9	6.0	30.9 (26–37)	94.0 (90–96)	1.15	**0.003**
Hyperaemia (*n* = 562)	82.4	31.5	82.4 (78–86)	68.5 (63–74)	1.16	**0.001**
Non-compressible (*n* = 520)	77.3	23.1	77.3 (72–82)	76.9 (71–82)	1.11	**0.029**
Absent luminal gas (*n* = 469)	42.6	8.0	42.6 (37–49)	92.0 (88–95)	1.13	**0.004**
Probe tenderness (*n* = 605)	96.9	56.5	96.9 (94–98)	43.5 (38–49)	1.18	** < 0.001**
Peri-appendiceal fluid (*n* = 539)	41.5	14.1	41.5 (36–47)	85.9 (81–90)	1.05	0.24
Peri-appendiceal echogenic fat (*n* = 627)	87.8	20.6	87.8 (84–90)	79.4 (75–83)	1.42	** < 0.001**

Bold represents significant *P*-values

*Appendicitis* simple or complicated appendicitis which is intraoperatively defined (except n = 2 CA patients who underwent conservative management only), *Other* negative appendicectomies which are histologically defined, and children who did not undergo appendicectomy, *Adj.* Adjusted, Odds ratio calculated using exp(multiple regression coefficient)

Sonographic signs which suggest CA over SA comprised a diameter above 10.1 mm (AUC 0.63, [95% CI: 0.57–0.69] presence of an appendicolith (*p* = 0.003) and the secondary sign of peri-appendiceal fluid (*p* = 0.004) (Table 3). When the two primary signs were grouped, the regression model correctly classified 73% of cases with AUC 0.68 [95% CI 0.60–0.75]. To diagnose CA over SA, we found USS had diagnostic accuracy of 82%, with sensitivity 52.2% [CI: 42.1–62.1] and specificity 96.4% [CI: 92.8–98.3].

**Table 3:** Utility of sonographic signs to differentiate complicated from simple appendicitis

Sonographic sign	CA (*n* = 113) % *n* (%)	SA (*n* = 226) % *n* (%)	Sensitivity %, (95% CI)	Specificity %, (95% CI)	AOR	*P*-value
Blind ending visualised (*n* = 286)	78.0 (%)	94.1	78.1 (68–86)	5.9 (3–10)	0.71	** < 0.001**
Appendicolith (*n* = 272)	47.1	23.5	47.1 (37–58)	76.5 (70–82)	1.15	**0.003**
Hyperaemia (*n* = 295)	75.0	85.7	75.0 (65–83)	14.3 (10–20)	0.90	0.4
Non-compressible (*n* = 269)	69.9	80.7	69.9 (59–79)	19.4 (14–26)	0.92	0.5
Absent luminal gas (*n* = 244)	32.0	47.3	32.0 (23–43)	52.7 (45–60)	0.91	0.06
Probe tenderness (*n* = 288)	95.4	97.5	95.4 (89–98)	2.5 (1–6)	0.59	**0.04**
Peri-appendiceal fluid (*n* = 277)	53.5	36.1	53.5 (43–64)	63.9 (57–70)	1.16	**0.004**
Peri-appendiceal echogenic fat (*n* = 302)	91.8	85.9	91.8 (85–96)	14.2 (10–20)	1.02	0.69

Bold represents significant *P*-values

Sensitivity and specificity calculated with Wilson-Brown 95% confidence intervals

*CA* Complicated appendicitis, *SA* Simple appendicitis, *AOR* Adjusted, Odds ratio calculated using exp (multiple regression coefficient)

**Fig. 3:**
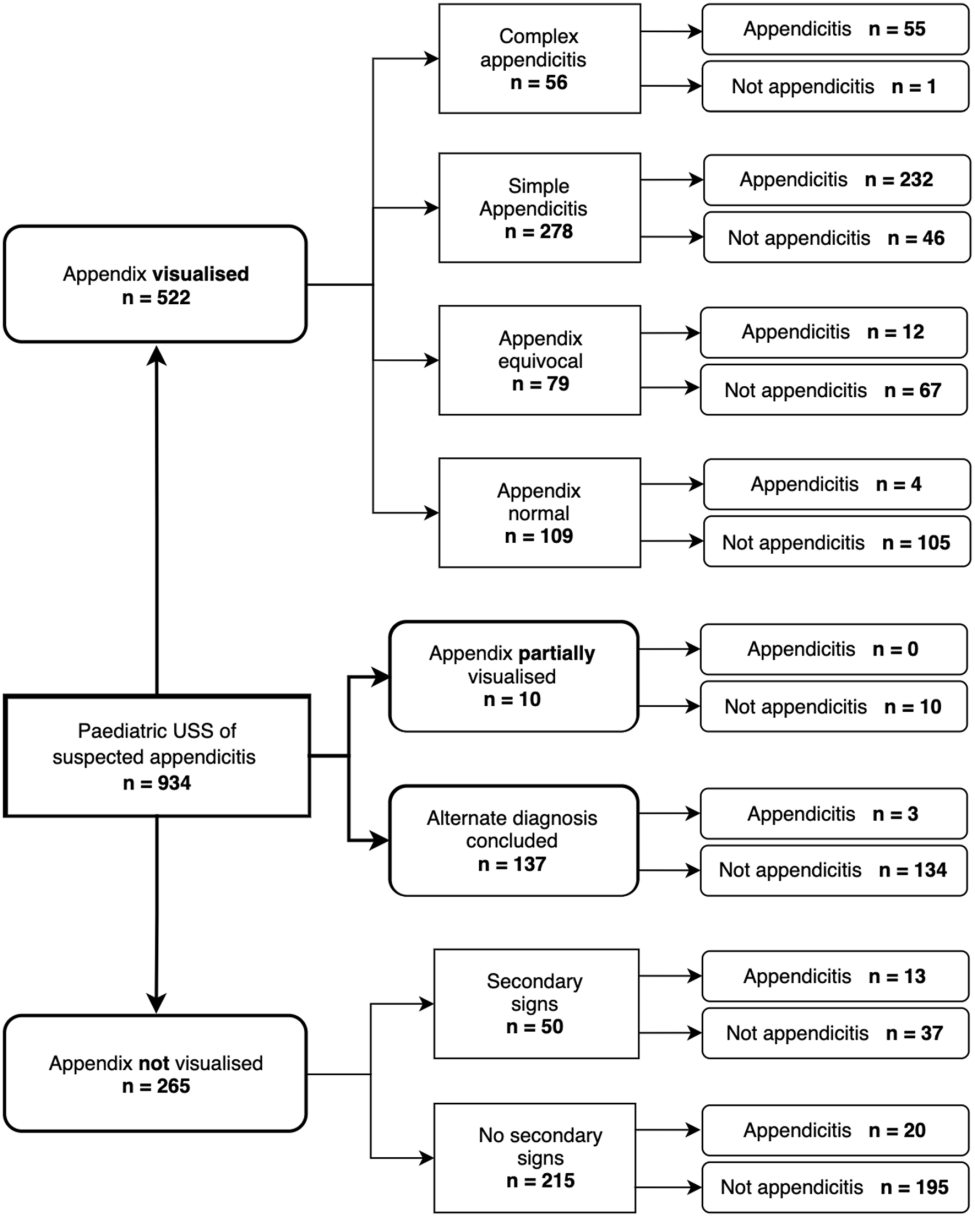
Flow diagram of ultrasound scan report conclusions with outcomes. *Appendicitis* = simple and complicated appendicitis which are intraoperatively defined (except *n* = 2 patients with complicated appendicitis who underwent conservative management only). *Not appendicitis* includes negative appendicectomies which are histologically defined, and children who did not undergo appendicectomy. *USS* Ultrasound Scan

Our sonographic worksheet was utilised in 83% of USS, with significantly lower use in the NegA group (71%) compared to other operative outcomes (*p* = 0.006). Conclusive USS reports matched the operative outcomes over 90% of the time (Fig. 3). A fifth of children (18%) who had an inconclusive USS had surgery (8% SA, 5% CA, and 5% NegA). When the appendix was not viewed, the presence of secondary signs did not often lead to surgery (26%). If neither the appendix nor secondary signs were viewed (*n* = 215), appendicitis was the diagnosis in 9% of cases.

## Discussion

Ultrasound scans are frequently performed in children with varying degrees of benefit in assisting the diagnosis of appendicitis. The sonographic signs are infrequently reported but have been shown to aid the diagnosis [9–23]. Our study aimed to investigate the diagnostic utility of USS by finding sonographic signs associated with appendicitis, and which differentiate SA and CA in children.

The negative appendicectomy rate was similar between the group who had USS and the group who did not (*p* = 0.18); we believe this is related to an excellent accuracy of clinician gestalt in diagnosing appendicitis in children presenting to our emergency department [2]. The time interval between USS and surgery can be an important factor in occurrence of perforation; different patient-dependent factors (e.g., need for resuscitation) and patient-independent factors (e.g., availability of the operating theatre and operating team) can prolong this interval, potentially affecting the occurrence of perforation in patients that are delayed to theatre. However, we found that the time interval between USS and surgery was slightly longer in the SA group compared to the CA group, although the difference was not statistically significant. Due to the retrospective nature of the study, we do not have an exact explanation for this finding but we speculate that children diagnosed with CA on USS were deemed to require surgery more urgently than children diagnosed with SA. In this respect, the USS might add to the clinical decision making in prioritising children with CA requiring urgent surgery.

### Appendix visualisation and ultrasound accuracy

Visibility of sonographic signs is markedly reduced if the appendix itself is not visualised. Our study had an overall 62% conclusive scan rate, closely linked to our overall 61% appendix visualisation rate. Studies of diagnostic accuracy of USS for paediatric appendicitis report a wide range of visualisation, with two Australian studies reporting 41% and 92% [5, 7]. Cundy et al. attribute their superior visualisation rate to diligent paediatric sonographers who employ multiple techniques and use tightly curved transducers more suited to smaller patients [6]. Reddan et al. were able to improve visualisation rates to 69% in a subsequent study, following sonographer training and implementation of a worksheet [13]. Aside from implementation of a worksheet, other institutional variables such as sonographer experience, type of hospital (regarding paediatric volume), and time of day also contribute [22]. Visualisation is also dependent on patient factors, such as duration of symptoms and their clinical presentation pre-USS. Patients who have a high suspicion of appendicitis could have developed more sonographic signs than clinically equivocal patients [7, 12, 22, 24].

Our study demonstrated appendiceal visualisation rates of 92% in SA, with only 48% NoA viewed. One explanation is that non-inflamed appendices are more difficult to visualise due to their smaller size and absent secondary features. However, our study included a higher appendicitis prevalence (36%) than Cundy et al. (28%) implying a smaller patient volume in our study but also suggesting consistent visualisation is still possible. Our CA visualisation rate (72%) was lower than SA, which we attribute to the difficulty delineating an appendix amongst associated inflammatory changes.

With non-visualisation of the appendix, secondary signs were present in 16% of USS, well within the range of 5–23% reported in the literature [10, 24–26]. In our cohort, a quarter of this group had appendicitis (26%), and 69% of these were CA. This relatively low rate of USS diagnosed appendicitis compared to Partain et al. (42%), is likely due to their stricter definition of a secondary sign including a ‘significant amount of fluid’ rather than our ‘peri-appendiceal fluid’, which is often physiological [12]. However, Held et al. report a 17% appendicitis rate, likely explained by the majority of their USS being in the non-visualised category (76%) [10]. When neither the appendix nor secondary signs were viewed, our appendicitis rate was within the range of previously reported values 2–9% [10, 12, 24]. It has been suggested that these children can be observed or discharged with increased confidence, although it remains case dependent.

Overall, our reported accuracy was inferior to previous meta-analyses reporting pooled sensitivity (88–89%) and specificity (94–97%) [27, 28]. However, this included heterogenous data from 12,926 children from different institutions. For example, some centres excluded non-diagnostic USS from analysis, which overestimate the true accuracy. For diagnosing CA over SA, our sensitivity was higher than previously reported values of 23–44%, with specificity similar at 93–100% [17–19, 29].

Our sonographic features were analysed using an established institutional worksheet. This provides sonographers with a conventional framework to ensure a systematic and thorough examination and ensures a standardised outcome source which increases the quality of our retrospective study. The application of a standardised worksheet is lacking in existing published literature [9, 10, 12, 19–21]. Therefore, their retrospective data are limited to sonographic signs that radiologists report, which means the relevant absence of signs may not be detailed.

### Primary sonographic signs suggesting appendicitis

The maximal appendiceal diameter is the most commonly studied sonographic sign. Our service uses 6 mm as a threshold, (sensitivity 97%, specificity 69%). However, our analysis suggests 7 mm is more accurate (sensitivity 86%, specificity 86%), particularly in the NegA group. This is similar to other studies, with some suggesting three categories [30–33]. We suggest balancing the statistical superiority of a more specific 7 mm threshold with the clinical risk of false negatives.

An appendicolith was a significant predictor of appendicitis in our cohort with specificity 94% (adjusted OR 1.15, *p* = 0.003), similar to Partain et al. (adjusted OR 7.9 [95% CI 1.7–37.2]) and Telesmanich et al. (OR 15.8 (*p* = 0.03)), with Trout et al. finding no association [12, 14, 15]. An appendicolith has been generally associated with failure of non-operative management; however, none of the “appendicolith-positive” patients in our series (6%) developed appendicitis after 12 months follow-up; a longer follow-up will be needed to confirm this finding.

Hyperaemia on doppler was most frequently seen in SA (86%), and significantly associated with appendicitis. This is consistent with most previous studies [12, 15, 34, 35].

Non-compressibility on probe pressure significantly predicted appendicitis, and had increased prevalence in SA. Potentially CA may have been decompressed with a perforation at the time of USS, or the appendiceal structure could be distorted amongst heterogenous inflammatory echoes. This sign is uncommonly reported in the literature [15].

Absence of luminal gas was significantly associated with appendicitis and most frequent in SA. There is a paucity of reported data studying this sign, with our data suggesting it has utility to rule in appendicitis (specificity 92%), with its absence having little value (sensitivity 43%).

### Secondary sonographic signs suggesting appendicitis

Focal pain with transducer pressure is uncommonly studied due to its similarity to palpation [11]. However, we found significant association with appendicitis, present in 97%, and probe tenderness carried the highest sensitivity (97%) and lowest specificity (44%) of all the sonographic signs. Its relative frequency in children who had a NegA (92%), could suggest clinicians value clinical right iliac fossa tenderness for diagnostic evaluation.

Peri-appendiceal fluid was not significantly associated with SA, consistent with the literature [9, 11, 13–15, 35]. However, other studies reported significance of right lower quadrant fluid, with some additionally finding free fluid [10, 12, 35]. This emphasizes the importance of location, volume and character descriptors, as free fluid can be physiological in small amounts, whereas heterogenous fluid can suggest pathology.

The presence of peri-appendiceal echogenic fat was most associated with appendicitis in our cohort, which is consistent with the published literature [11, 14, 15, 35].

### Primary sonographic signs predicting complicated over simple appendicitis

The statistically optimal threshold maximal appendix diameter was 10.1 mm for CA (sensitivity 54%, specificity 75%), which is larger than previously studied (9 mm) [21]. An appendicolith was a significant predictor of CA, which has been reported previously [16, 17, 20, 21, 23]. Visualisation of a blind-ending structure is included as a confirmatory variable on our worksheet to ensure viewing of the correct anatomy. It was negatively associated with CA which could be due to difficulty identifying the appendix tip amongst inflammatory tissue. Hyperaemia, non-compressibility, and absent luminal gas were not associated with CA in our study although some previous studies have found them to be helpful [17, 22].

### Secondary sonographic signs predicting complicated over simple appendicitis

Probe tenderness was negatively associated with CA, which was unexpected with unclear clinical significance. It has not been previously studied in the literature and reflects a subjective, non-specific yet sensitive sign of CA. Peri-appendiceal fluid was detected most frequently and significantly associated with CA, although it was also present in both SA and children without appendicitis. Heterogenous fluid has been reported as the most predictive sign for CA [17, 20, 22, 23]. We hypothesize this finding reflects perforation with leaked intra-luminal content or inflammatory exudate, and should be further characterised in a sonographic worksheet or report. Echogenic fat was not associated with CA as it was also common in SA, consistent with most previous studies [16, 17, 20–23].

### Strengths and limitations

Strengths of our study include a large study population over a relative short period and the use of a standardised worksheet, reducing the variability in reporting, and ensuring accurate data collection. Limitations of our study stem from its retrospective design. Our literature review revealed other sonographic signs reported to be associated with appendicitis, such as appendiceal mural thickness and bowel signs which were not assessed in our study. Peri-appendiceal fluid is one of the signs found to be good differentiator between SA and CA; however, our study did not specifically address if it was simple or heterogeneous; we plan to add this information to our worksheet in the future. Investigating inter-observer reliability between sonographers or radiologists was another potential limitation. This would have allowed the operator-dependence of USS to be quantified and whether experience of sonographers improved their performance. Additional patient data such as body mass index, clinical presentation details, time from onset of symptoms to USS, and complication rates could enable a thorough investigation into factors affecting USS accuracy. It would also be useful to explore the clinician’s views about USS and appendicectomy, allowing us to gauge their perceived utility and how certain findings influence their management.

## Conclusions

We identified sonographic features which were significantly associated with simple and complicated appendicitis. We recommend institutions adopt a standard sonographer worksheet to facilitate comprehensive examination and increase the clinical utility of ultrasound.

### Supplementary Information

Below is the link to the electronic supplementary material.

Supplementary file1 (PDF 116 KB)

## Data Availability

The datasets generated during and/or analysed during the current study are available from the corresponding author on reasonable request.
