# Supplementary material for: The utility of sonographic signs to diagnose simple and complicated appendicitis in children
Source: Pediatr Surg Int. 2023 Feb 11;39(1):114. doi: 10.1007/s00383-023-05397-y (PMC9918567; doi:10.1007/s00383-023-05397-y)
Supplement: Supplementary file 1 — Supplementary file1 (PDF 116 KB) [file 383_2023_5397_MOESM1_ESM.pdf]

SUPPLEMENTARY INFORMATION

Sonographer Worksheet

THE UTILITY OF SONOGRAPHIC SIGNS TO DIAGNOSE SIMPLE AND COMPLEX APPENDICITIS IN CHILDREN

Pediatric Surgery International

Lauren Tong[1], Ramesh M Nataraja[1,2,3], Keith VanHaltren[4], Tania H Sulaksana[3], Toby I Vinycomb[3], Maurizio Pacilli[1,2,3]

Affiliations:

[1]Department of Paediatrics and [2]Department of Surgery, School of Clinical Sciences at Monash Health, Faculty of Medicine, Nursing and Health Sciences, Monash University, Melbourne, Victoria, Australia.

[3]Department of Paediatric Surgery, Monash Children's Hospital, Melbourne, Victoria, Australia.

[4]Department of Paediatric Radiology, Monash Children's Hospital, Melbourne, Victoria, Australia.

Corresponding author:

Mr. Maurizio Pacilli MBBS (Hons), MD (Res), FRCS (Paed Surg)

Department of Paediatric Surgery

Level 5, Monash Children's Hospital

246 Clayton Road, Clayton, VIC 3168, Australia

T: +61385723837 F: +61385723007

Email: maurizio.pacilli@monash.edu

ORCID iD: 0000-0003-1259-4304

Paediatric Abdomen (Limited)

Clinical History ……………………………………………………………………………………………………

** This is a focussed examination, clinically guided by the clinical indications on the referral and the clinical assessment at the time of the examination*

All examinations

Right Kidney hydronephrosis (No / Yes) ………………… **Left Kidney** hydronephrosis (No / Yes) ……………………

Free Fluid (none, mild, moderate, severe) Location/s …………………… **SMA / SMV** Typical anatomy (Yes / No)

Gallbladder Normal (Yes / No / Not fasted) ……………………………………………………………………

Ovaries are to be examined if a female + lower abdomen pain + normal study **Rt Ov.** Normal (Yes / No) **Lt Ov.** Normal (Yes / No)

Comment ………

Appendix: Examined (No / Yes) Abnormal: (No / Yes / Equivocal) - Blind ending seen (Yes / No)

Seen / Not seen (Max. AP diam) …….. mm (* > 6mm Abnormal) Focal pain (over appendix) (mild, mod, severe)

Circle any of the following if present Appendicolith, Peri-appendiceal fluid, Peri-appendiceal echogenic fat, Hyperaemia, Non-compressible, No luminal gas

Location if seen *(circle & draw)* McBurneys, Inferior, Pelvis, Retro-caecal, Rt Paracolic (lat to caecum). Other ……………

Comment ………………………………………………………………………………………………

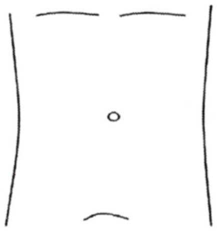

Intussusception Examined (No / Yes) Present: (No / Yes / Equivocal)

If yes … Location ………………….. Length ……… cm *(draw)*

Interloop fluid (>9mm) : (No / Yes) Colour (Present / Reduced / Absent) ……………………………………

Pylorus- Examined (No / Yes) Sterile Water (requirement) (Yes / No) Hypertrophic pyloric stenosis (Yes / No / Equivocal)

Pylorus Length ………… (mm) Wall thickness ……… (mm) *(*Length > 17mm abnormal, Wall thickness >3mm abnormal)*

Transit of fluid: Comment ……………………………………………………………………………………

Lymph nodes seen - (No / Yes) If yes (circle) … RIF, LIF, Right Mid, Left Mid, Paraaortic ……………

Approx. No ………… Max. AP diameter ………… cm Largest (mm) ……. x ……... x ..…. cm Focal Pain (No / Yes)

Comment ……………………………………………………………………………………………………

COMMENT & Other findings ………………………………………………………………………………

………..

………..

Key images are …………… Key clips are ………… (Radiologist to consider hyperlinking in the CSH PACs report)

Sonographer (1) ………… **Sonographer (2)** MonashHealth **Duty Sonologist**………

Sonographer (1) ………… Discussed with …………… Discussed with ………

Scanned with…… (if ltd. region)……………………… Scanned with …………

NOTE: This is a sonographer's worksheet NOT a consultant's report. It documents a sonographer's impressions during the examination and complements images captured during the study. The consultant's report may vary from the worksheet comments. The REPORT must be reviewed prior to formulation of any management strategy
